# Medical tourism in Malaysia: how can we better identify and manage its advantages and disadvantages?

**DOI:** 10.3402/gha.v7.25201

**Published:** 2014-09-10

**Authors:** Meghann Ormond, Wong Kee Mun, Chan Chee Khoon

**Affiliations:** 1 Cultural Geography Chair Group, Wageningen University, Wageningen, The Netherlands; 2 Faculty of Business and Accountancy, University of Malaya, Kuala Lumpur, Malaysia; 3 Centre for Population Health, Department of Social and Preventive Medicine, Faculty of Medicine, University of Malaya, Kuala Lumpur, Malaysia

**Keywords:** medical tourism industry, statistical data, destination countries, healthcare commodification, public–private health care investment, entrepreneurial state, Southeast Asia

## Abstract

Following the identification of medical tourism as a growth sector by the Malaysian government in 1998, significant government sector and private-sector investments have been channeled into its development over the past 15 years. This is unfolding within the broader context of social services being devolved to for-profit enterprises and ‘market-capable’ segments of society becoming sites of intensive entrepreneurial investment by both the private sector and the state. Yet, the opacity and paucity of available medical tourism statistics severely limits the extent to which medical tourism's impacts can be reliably assessed, forcing us to consider the real effects that the resulting speculation itself has produced and to reevaluate how the real and potential impacts of medical tourism are – and should be – conceptualized, calculated, distributed, and compensated for. Contemporary debate over the current and potential benefits and adverse effects of medical tourism for destination societies is hamstrung by the scant empirical data currently publicly available. Steps are proposed for overcoming these challenges in order to allow for improved identification, planning, and development of resources appropriate to the needs, demands, and interests of not only medical tourists and big business but also local populations.

Proponents generally envision how medical tourism – ‘all the activities related to travel and hosting a tourist who stays at least one night at the destination region, for the purpose of maintaining, improving or restoring health through medical intervention’ (1) – can be used by destinations to attract foreign exchange, mitigate health worker brain-drain, and improve health care and tourism infrastructure (2). Correspondingly, medical tourism has been actively embraced by governments and private-sector actors in a growing number of lower- and middle-income countries as a potentially powerful economic growth engine. Meanwhile, critics generally warn that medical tourism may harm destinations by stimulating private health care development unresponsive to locals’ needs and resources (3). Yet, although more scholars, governments, and medical bodies in source countries are calling attention to how medical tourism may adversely impact both source and destination societies (4), there has been relatively little outspoken resistance within destination countries to it.

Those engaging in contemporary debate over the current and potential benefits and adverse effects of medical tourism for destination societies generally turn to sorely inadequate government-reported medical tourism statistics – widely acknowledged to deploy opaque definitions and creative counting practices – to support their arguments (5). These statistics render medical tourism's actual volumes and contributions so difficult to gauge that the many estimates and claims made by those on either side of the debate should be treated with caution (6).

In Malaysia, one of Southeast Asia's most prominent medical tourism destinations, national medical tourism statistics derive from the reported numbers of all foreign patients treated by Malaysian Healthcare Travel Council–endorsed medical facilities and their associated revenue. These are published only at the national level, do not include all medical facilities, and do not recognize or measure medical tourism's diverse subnational direct and indirect economic and social impacts. Furthermore, available data indiscriminately encompass all registered patients with a foreign passport, which by default also encompass expatriates, migrants, business travelers, and holiday-makers for whom health care may not be the main motive for their stay (7–9). The opacity and paucity of available figures, therefore, severely limits the extent to which we can reliably quantify medical tourism's impacts in Malaysia. This quantitative void forces us both to consider the real effects that the speculation this void has itself produced and to reevaluate how the real and potential impacts of medical tourism are – and should be – conceptualized, calculated, distributed, and compensated for.

In this brief debate piece, we use the lenses of our diverse engagements with the Malaysian government, medical tourism industry, and health care providers and users to relate how medical tourism statistics have been used in Malaysia and reflect on the limitations of these framings. We then identify steps to more productively advance the discussion about the challenges and benefits of medical tourism.

## Proponents’ perspective

The Malaysian government identified medical tourism as a growth sector during the 1997–98 Asian financial crisis, when significant numbers of Indonesians began to turn to Malaysian private hospitals for affordable, quality health care. In the following decade, private hospitals – concentrated mainly in Penang, Melaka, Selangor, Sarawak, and Johor – worked alongside and through their respective state governments; private hospital associations; and the Malaysian Ministries of Health, Tourism, and Trade and Industry. They sought to attract not only neighboring Indonesians – characterized by high volumes yet low per patient expenditure – but also higher-spending medical tourists from further afield (e.g. Australia, Bangladesh, China, India, Japan, Nepal, the United Kingdom, the United States, and the Middle Eastern countries) (7, 10, 11). The Malaysian government's investment tax allowance further spurred private health care facilities promoting medical tourism to invest in internationally recognized accreditation schemes (e.g. Joint Commission International and Malaysian Society for Quality in Healthcare) and state-of-the-art medical equipment in order to develop technology-intensive private health care facilities and ensure ‘world-class’ care standards considered necessary to attract medical tourists (12, 1).

With the 2010 launch of the Economic Transformation Program (ETP), intended to transform Malaysia into an upper middle-income country with a knowledge-based economy, interest in harnessing medical tourism's economic potential grew. The ETP earmarked health care as one of the country's 12 National Key Economic Areas (NKEAs) deemed to have the potential to spur growth (13). Part of the health care NKEA, medical tourism is intended to generate MYR 9.6 billion^1^ in revenue and MYR 4.3 billion in gross national income and to require 5,300 more medical professionals by 2020 (14). For-profit hospitals are expected to invest MYR 335 million in hospital infrastructure in order to be prepared for 1.9 million foreign patients annually by 2020 (14). Despite the specificity of these targets, however, scant empirical data are publicly available to evaluate whether these targets are being met and, indeed, even the basis for such projections.

Medical tourism is believed to be contributing to the national economy. Government-reported revenue from medical tourism in Malaysia amounted to MYR 683 million – 9% above its 2013 target (15). Although this added only 0.1% to Malaysia's MYR 985 billion gross domestic product (GDP) in 2013 (16), medical tourism's year-on-year double-digit growth is being used to attract foreign investment and joint ventures in the Malaysian health care industry (e.g. the 2013 Ramsay Sime Darby Healthcare joint venture (17)). Large Malaysian health care conglomerates also plan to reap economic gains from medical tourism despite its current limited contribution. For example, although medical tourism contributed only 4% (MYR 67 million) to KPJ Healthcare Bhd's 2013 overall revenue, KPJ expects this to rise to 25% by 2020 by more intensively promoting its Malaysian facilities (15, 18).

Although growing numbers of Malaysian health care facilities are actively promoting medical tourism, some 95% of Malaysian private hospitals’ clientele is reported to be Malaysian (19). Private and corporatized hospitals’ medical tourism revenue, therefore, is viewed as helping to not only sustain but also upgrade these facilities to local private health care users’ benefit, providing Malaysians with alternatives to crowded public health care provision. Investment in medical tourism infrastructure is furthermore considered to generate demand for goods and services in allied sectors (e.g. clinical research and development, pharmaceuticals, and medical equipment) (14). Transport, retail, commercial care, and hospitality sectors can also benefit from spending by medical tourists and their companions, generating medical- and non-medical jobs and spurring the growth not just of large but also small and medium enterprises (1, 10). Local businesses in Kuching, Melaka, and Johor Bahru, for example, are seen to be flourishing with the influx of cross-border Indonesian and Singaporean medical tourists. However, to date, no empirical evidence is available on the multiplier effect of medical tourism on other sectors and local economies. Such data would help to elucidate the effectiveness of government investment in the growth of the medical tourism industry.

## Critics’ perspective

Malaysia's development as a medical tourism destination has unfolded within a context of health care corporatization and privatization that has profoundly transformed the country's health care landscape and horizons (20, 21). Critics see medical tourism as an expression of health care commodification, highlighting the Malaysian state's multiple roles as funder and provider of public-sector health care, regulator, and pre-eminent investor in commercial health care.

Medical tourism is embedded in a broader political economy in which social services have been devolved to for-profit enterprise and ‘market-capable’ segments of society have become sites of intensive entrepreneurial investment by both the private sector and the state (7). Critics note, for example, that, although private hospitals account for approximately 30% of all hospital admissions (22), government-linked companies at both federal and provincial levels currently control more than 40% of commercial hospital beds in Malaysia (23). Among the country's most prominent hospitals endorsed for medical tourism are for-profit hospitals belonging to the Johor State Government–owned KPJ chain and the IHH Healthcare Bhd–owned Pantai and Gleneagles chains. IHH, the world's second-largest listed health care operator based on market capitalization, is majority-owned by the Malaysian government's sovereign investment arm, Khazanah (24). Both KPJ and IHH command ever-larger slices of the Asian health care market both through their acquisition of regional hospitals (e.g. KPJ's acquisitions in Indonesia and Bangladesh) and their promotion of medical tourism in Malaysia.^2^

This novel situation is perceived to be rife with conflicts of interest and divergent priorities (7, 20, 21). Although health care is not inscribed in the Malaysian constitution as a right, Malaysian nationals have become accustomed to *de facto* entitlement to publicly provided and highly subsidized health care since decolonization in 1957. Citizens may or may not avail themselves of this universalist entitlement, yet even those who do not do so still benefit from its second-order effects. The availability of publicly provided health care (of a certain quality) acts as a restraining price bulwark that helps to keep private health care charges within a more affordable range. With the state's increasing stakes in commercial health care however, will there be a benign neglect of the public sector as the state encourages those who can afford it to migrate to the private sector for their healthcare needs? This could further entrench a two-tier health care system, with deluxe priority care for the better-off (including ‘medical tourists’) and a rump, underfunded public sector for the rest (25, 26).

Might Malaysians, however, benefit indirectly from profits accruing to the public purse from medical tourism and other for-profit healthcare investments? The Malaysian national oil company Petronas’ total equity is approximately ten times that of IHH. Comparing the MYR 87.8 million that IHH paid in corporate taxes to the Malaysian government in FY2011 with the MYR 66.3 billion in taxes and dividends generated by Petronas in FY2012 suggests modest returns on IHH's healthcare investments (27, 28). As for where revenue derived from medical tourism goes (e.g. special taxation regimes, economic leakages), little is known, and discussions about corporate accountability are absent.

## Conclusions

The debate over the gains and adverse effects of ‘medical tourism’ is far from Malaysia-specific. Rather, it is a concern shared by medical tourism destinations more generally (as in Israel (29) and Costa Rica (30)). However, given the great margin of uncertainty over basic data about medical tourism, conclusions on both sides are unavoidably speculative. Indeed, it is possible to assume – as we have shown here – diametrically opposed positions on the issue with little prospect of resolution.

There is much work to be done in order to better grasp medical tourism's actual impacts on destinations. This first requires us – as policymakers, industry actors, scholars, citizens, and consumers – to acknowledge not only medical tourism's imbrication in a broader range of transnational care pursuits and provision but also medical tourism's articulation in a broader (and increasingly global) political economy of health care. This would allow us to start asking more astute questions about the ways in which different stakeholders conceptualize ‘medical tourism’ and to begin to measure variables that enable analyses that transcend disembodied claims about growth (31).

This also requires us to acknowledge that medical facilities and the diverse communities in which they are inserted receive different volumes and types of foreign patients that may or may not be ‘medical tourists’, with their own unique needs, wants, socioeconomic and political statuses, and spending patterns. To better respond to this *de facto* diversity, the knowledge we produce about medical tourism must be useful to identify, plan for, and develop resources appropriate to the needs, demands, and interests of not only medical tourists and big business but also local populaces.

## Footnotes

1 The current exchange rate is USD 1=MYR 3.2.

## Footnotes

2 Currently, however, non-government-linked companies (e.g. Island Hospital and Penang Adventist Hospital) attract the lion's share of medical tourism to Malaysia.

## References

[1] Musa G, Doshi D, Wong KM, Thirumoorthi T (2012). How satisfied are inbound medical tourists in Malaysia? A study of private hospitals in Kuala Lumpur. J Trav Tour Mark.

[2] Bookman M, Bookman K (2007). Medical tourism in developing countries.

[3] Sengupta A (2011). Medical tourism: reverse subsidy for the elite. Signs.

[4] Snyder J, Dharamsi S, Crooks VA (2011). Fly-By medical care: conceptualizing the global and local social responsibilities of medical tourists and physician voluntourists. Global Health.

[5] Connell J (2011). Medical tourism.

[6] Wong KM, Musa G, Hall CM (2012). Medical tourism in Asia: Thailand, Singapore, Malaysia, and India. Medical tourism: the ethics, regulation, and marketing of health mobility.

[7] Ormond M (2013). Neoliberal governance and international medical travel in Malaysia.

[8] Malaysia Healthcare Travel Council (2013). Statistics. http://www.mhtc.org.my/en/statistics.aspx.

[9] Ministry of Health Malaysia (2014). Health facts 2014. http://www.moh.gov.my/images/gallery/publications/HEALTHFACTS2014.pdf.

[10] Ormond M, Sulianti D (2014). More than medical tourism: lessons from Indonesia and Malaysia on South-South intra-regional medical travel. Curr Issues Tour.

[11] Malaysia Healthcare Travel Council (2013). The first Medical Tourism Concierge and Lounge in Malaysia. http://www.mhtc.org.my/en/press-release.aspx.

[12] Johnston R, Crooks VA, Snyder J, Kingsbury P (2010). What is known about the effects of medical tourism in destination and departure countries? A scoping review. Int J Equity Health.

[13] Pemandu (2010). Economic transformation programme: a roadmap for Malaysia. http://etp.pemandu.gov.my/download_centre.aspx.

[14] Pemandu (2010). Creating wealth through excellence in healthcare. http://www.moh.gov.my/images/gallery/ETP/NKEAPenjagaanKesihatan.pdf.

[15] Pemandu (2013). ETP annual report, 2013. Healthcare. http://etp.pemandu.gov.my/annualreport2013/upload/ENG/14_NKEA12_ENG_HC.pdf.

[16] Bank Negara Malaysia (2014). National accounts (GDP) at current prices. http://www.bnm.gov.my/index.php?ch=statistic_nsdp.

[17] Lee L (2013). Ramsay JV aims to have at least 12 hospitals in three to five years. The Star.

[18] Teo R (2014). KPJ sees increasing contributions from Malaysian, medical tourism sectors. The Borneo Post.

[19] To Your Health [radio programme] (2014). The Breakfast Grille.

[20] Chan CK (2010). Re-inventing the welfarist state? The Malaysian health system in transition. J Contemp Asia.

[21] Chee HL (2010). Medical tourism and the state in Malaysia and Singapore. Glob Soc Policy.

[22] Ministry of Health Malaysia (2013). Health facts 2013. http://www.moh.gov.my/images/gallery/publications/HEALTHFACTS2013.pdf.

[23] Chan CK (2014). The Malaysian health system in transition: the ambiguity of public and private.

[24] IHH (2014). Overview. http://ihh-healthcare.com/hospital-overview.php.

[25] Chan CK, Ooi KB, Goh BL (2010). Re-thinking the state and healthcare in Penang. Pilot Studies for a New Penang.

[26] Chan CK (2006). What's new in the Arusha statement on *New Frontiers of Social Policy?*. Glob Soc Policy.

[27] IHH (2012). IHH undertakes first ever concurrent IPO in Malaysia and Singapore. http://ihh.anticsstudios.com/files/press-release/EnglishPressRelease_IHHIPOLaunch_3July.pdf.

[28] Petronas (2012). Petronas annual report 2012. http://www.petronas.com.my/investor-relations/Pages/annual-report.aspx.

[29] Zehavi A, Zer N (2013). A public road to globalization: public services and customer internationalization in Israel. Admin Soc.

[30] Lee CA (2012). Health care at a crossroads: medical tourism and the dismantling of Costa Rican exceptionalism.

[31] Pocock NS, Phua KH (2011). Medical tourism and policy implications for health systems: a conceptual framework from a comparative study of Thailand, Singapore and Malaysia. Global Health.

